# Survival prediction and prognostic factors in colorectal cancer after curative surgery: insights from cox regression and neural networks

**DOI:** 10.1038/s41598-023-42926-0

**Published:** 2023-09-21

**Authors:** Shayeste Alinia, Mohammad Asghari-Jafarabadi, Leila Mahmoudi, Solmaz Norouzi, Maliheh Safari, Ghodratollah Roshanaei

**Affiliations:** 1 Department of Statistics and Epidemiology, School of Medicine, Zanjan University of Medical Sciences, Mahdavi Blvd, Zanjan, 4513956111 Iran; 2 Faculty of Health, Road Traffic Injury Research Center, Tabriz University of Medical Sciences, Golgasht St. Attar E Neshabouri St., Tabriz, 5166614711 Iran; 3 Cabrini Research, Cabrini Health, Malvern, VIC 3144 Australia; 4 Faculty of Medicine, Nursing and Health Sciences, School of Public Health and Preventative Medicine, Monash University, Melbourne, VIC 3004 Australia; 5 Department of Psychiatry, Faculty of Medicine, Nursing and Health Sciences, School of Clinical Sciences, Monash University, Clayton, VIC 3168 Australia; 6 Department of Biostatistics, School of Medicine, Arak University of Medical Sciences, Arak, Iran; 7 Department of Biostatistics, Modeling of Non-Communicable Diseases Research Center, School of Public Health, Hamadan University of Medical Sciences, Hamadan, Iran

**Keywords:** Cancer, Gastroenterology, Medical research, Risk factors, Mathematics and computing

## Abstract

Medical research frequently relies on Cox regression to analyze the survival distribution of cancer patients. Nonetheless, in specific scenarios, neural networks hold the potential to serve as a robust alternative. In this study, we aim to scrutinize the effectiveness of Cox regression and neural network models in assessing the survival outcomes of patients who have undergone treatment for colorectal cancer. We conducted a retrospective study on 284 colorectal cancer patients who underwent surgery at Imam Khomeini clinic in Hamadan between 2001 and 2017. The data was used to train both Cox regression and neural network models, and their predictive accuracy was compared using diagnostic measures such as sensitivity, specificity, positive predictive value, accuracy, negative predictive value, and area under the receiver operating characteristic curve. The analyses were performed using STATA 17 and R4.0.4 software. The study revealed that the best neural network model had a sensitivity of 74.5% (95% CI 61.0–85.0), specificity of 83.3% (65.3–94.4), positive predictive value of 89.1% (76.4–96.4), negative predictive value of 64.1% (47.2–78.8), AUC of 0.79 (0.70–0.88), and accuracy of 0.776 for death prediction. For recurrence, the best neural network model had a sensitivity of 88.1% (74.4–96.0%), specificity of 83.7% (69.3–93.2%), positive predictive value of 84.1% (69.9–93.4%), negative predictive value of 87.8% (73.8–95.9%), AUC of 0.86 (0.78–0.93), and accuracy of 0.859. The Cox model had comparable results, with a sensitivity of 73.6% (64.8–81.2) and 85.5% (78.3–91.0), specificity of 89.6% (83.8–93.8) and 98.0% (94.4–99.6), positive predictive value of 84.0% (75.6–90.4) and 97.4% (92.6–99.5), negative predictive value of 82.0% (75.6–90.4) and 88.8% (0.83–93.1), AUC of 0.82 (0.77–0.86) and 0.92 (0.89–0.95), and accuracy of 0.88 and 0.92 for death and recurrence prediction, respectively. In conclusion, the study found that both Cox regression and neural network models are effective in predicting early recurrence and death in patients with colorectal cancer after curative surgery. The neural network model showed slightly better sensitivity and negative predictive value for death, while the Cox model had better specificity and positive predictive value for recurrence. Overall, both models demonstrated high accuracy and AUC, indicating their usefulness in predicting these outcomes.

## Introduction

Colorectal cancer (CRC) is a devastating disease with high mortality rates and is the third leading cause of death worldwide^1^. While the incidence rate of CRC varies across different regions of the world, it is more prevalent in American and European countries compared to Middle Eastern countries^2^. Unfortunately, the incidence of CRC is increasing in low and middle-income countries, such as Iran, due to the rising prevalence of modifiable risk factors, including sedentary lifestyles and improper diet^3,4^.

To control the burden of CRC, it is crucial to improve both modifiable and non-modifiable risk factors^5^. The good news is that CRC is one of the most curable cancers when detected at an early stage^6^. Therefore, the primary goal of follow-up programs after colorectal cancer treatment is to increase the survival rate by detecting recurrence in the early stages of the disease, allowing for more curative surgeries and increasing the probability of patient survival^7^. As a result, there is a pressing need to conduct a study to identify the factors affecting the recurrence and survival of patients with colorectal cancer.

Survival analysis is a popular statistical technique used to explore the correlation between explanatory variables and response variables in the context of time-to-event data^8^. While the Cox regression model is a widely accepted and commonly employed method in survival analysis, recent studies have shown that neural network-based analytical methods have become increasingly popular due to their ability to capture complex model relationships and improve learning performance, resulting in better diagnostic and prediction accuracy^9^.

In this study, we aim to compare the predictive ability of a neural network model with that of the Cox regression model in analyzing the survival rates of patients with colorectal cancer after curative surgery. Our goal is to utilize this advanced machine learning technique to improve our understanding of the complex relationships between patient characteristics and outcomes, ultimately improving patient care and prognosis.

## Materials and methods

### Study population and data collection

This study is a retrospective cohort of 284 colorectal cancer patients with resection referred to Imam Khomeini clinic in Hamadan during 2001–2017. In the present study, out of the 284 patients who were enrolled, 131 individuals encountered disease recurrence and 121 patients passed away during the follow-up duration. The recurrence of the disease was evaluated by considering the time gap between the surgery and the next instance of cancer recurrence. The patients who expired during the study were treated as censored observations. To obtain information regarding these cases, their contact information was collected, and a telephone follow-up was conducted to document their status in the records. All participants in the study or their legal guardians provided written informed consent prior to registration in the study, and all procedures were performed in accordance with relevant guidelines and regulations. Briefly, all demographic and clinical (pathological) information were extracted from the patient's files, the primary outcomes were recurrence and time to recurrence, death and time to death. The studied variables include age at the time of diagnosis (years), gender (female:1; male:2), Body Mass Index (BMI: kg/m2), and clinical/pathological variables such as metastasis to other sites (no:0; yes:1), cancer site (colon:1; rectum:2), surgery (no:0; yes:1), radiotherapy (no:0; yes:1), chemotherapy (no:0; yes:1), number of chemotherapy (0:no; 1: < 6; 2:6 +), morphology (0:no adeno; 1:adeno), grade (differentiation level) (1:well; 2:moderate; 3:poor), tumor size (1: < 4; 2: >  = 4 < 7; 3: =  > 7), disease stage(1:B; 2:C;3:D), PT-stage(1:T2; 2:T3; 3:T4; 4:Tx), and PN-stage(1:N2; 2:N3; 3:N4; 4:Nx).

### Statistical analysis

The data were analyzed using Stata (ver. 17, StataCorp, LLC, College station, Texas, USA), and R4.0.4 (https://cran.r-project.org) softwares. A significance level was considered 5%. The survival time of patients with colorectal cancer had calculated in months, and the data for categorical variables was reported as frequency and percentage. The median survival times of patients, both overall and specifically for those with recurrence, along with the median durations from the non-terminal event to the terminal event, were computed utilizing the Kaplan–Meier estimator. Besides we used this estimator to estimate the 1-, 3-, 5- and 10-year survival probabilities for the terminal event, the non-terminal events. The Cox regression model was employed to determine the factors affecting the survival time of patients. The assumption of proportional hazards was tested using Schoenfeld residual method^8^.

In the field of predicting postoperative mortality in healthcare, artificial neural network (ANN) and Cox regression models are used as the most common prediction models^10^. However, to evaluate and compare ANN and Cox models, few studies are conducted in terms of internal validity and external validity, which are considered as an evaluation for the performance measure of the models ^10^. One of the advantages of this method compared to the neural network is the simplicity and comprehensibility of the results^8,11^. The Cox model provides researchers with the capacity to assess the impact of multiple covariates, commonly referred to as predictor variables, on the hazard rate of an event. This is achieved while accounting for censoring^12^. To elaborate, in the context of a medical investigation exploring the lifespan of patients exposed to different therapeutic regimens, the Cox model readily supplies hazard ratios for each unique treatment. This enables medical professionals and researchers to comprehensively comprehend the relative effects of the interventions on patient prognosis^13^.

Also, due to the use of statistical distributions, this method can be used to investigate the simultaneous effect of several variables on survival time^8^. Furthermore, owing to the utilization of statistical distributions, this approach can be employed to examine the concurrent impact of multiple variables on survival duration^8^. This prominently facilitates the discernment of the individual effects of each variable on the hazard of the event while controlling for additional factors^14^. Within the framework of the Cox regression model, the identification of time-dependent variables is also feasible, enabling the capture of longitudinal fluctuations over time that may influence the likelihood of the focal event. Consequently, this model provides the capability to assess the concomitant effects of these time-dependent variables on the magnitude of risk associated with distinct variable constellations, even as temporal progression unfolds^15^.

Although the Cox regression model holds wide applicability in postoperative mortality prediction studies, there exist scenarios where the adoption of the Artificial Neural Network (ANN) model may be more fitting. Neural networks possess the ability to capture intricate and non-linear patterns, making them a superior choice in such contexts^16,17^. Comprising interconnected nodes (neurons) organized in layers, they excel at learning intricate patterns that could challenge conventional linear models^18^. ANNs tend to exhibit lower sensitivity to outliers compared to certain other statistical models. This attribute is a consequence of the network's architecture and optimization process, which enable it to adapt and fine-tune its parameters to the overall trends within the data. While outliers still hold influence, their impact on the model's predictions might not be as drastic as in simpler linear models^16,17^.

Cox regression yields interpretable hazard ratios and is particularly suitable when the assumption of proportionality is upheld^19,20^. In contrast, ANNs possess the potential to capture intricate relationships and might perform more effectively when confronted with non-linear or time-varying effects. A comprehensive examination of these facets enables a more thorough analysis of the two models^19–21^.

To train the artificial neural network (ANN) model, we first randomly divided our dataset into training and testing sets to prevent overfitting. The training set was used to teach the ANN model to recognize patterns in the data and make predictions, while the testing set was used to evaluate the accuracy of the model's predictions on new data. So, 70% of the data was used for training, and 30% of the data was used for testing the network.

Next, we performed a process of hyperparameter tuning to optimize the performance of the ANN model. This involved adjusting various parameters of the model, such as the number of hidden layers, the number of nodes in each layer, the learning rate, and the activation function, in order to find the best combination that resulted in the highest accuracy of the model. This process was carried out using a grid search method, in which multiple combinations of hyperparameters were tested, and the best-performing combination was selected based on the accuracy of the model on the testing set.

With different fits, a three-layer perceptron neural network was used, the first layer including 14 input variables. The middle layer has 5 nodes, the third layer, which is determined based on the type of response variable, has 2 nodes, learning rate was 0.01 and in hidden layers, activation functions were logistic, and the Backpropagation (Backward propagation of errors) algorithm was used to train artificial neural networks.

Diagnostic indices, including sensitivity, specificity, positive predictive value, accuracy, negative predictive value, and area under the receiver operating characteristic curve (ROC), with a 95% confidence interval (CI), were used to measure the quality and appropriateness of each.

### Ethical considerations

Ethics approval and consent to participate. The study protocol was approved by the institutional review board (IRB) of Tabriz University of Medical Sciences (ethics code: IR.TBZMED.REC.1400.457).

## Results

### Participants

In this study, 284 participants with resected colorectal cancer (CRC) were analyzed. Of these, 134 patients (47.1%) were women. The study found that 121 (42.6%) patients died, and 131 (46.1%) patients had a recurrence. Only 16 (19.8%) of the patients with recurrence survived until the end of the study. Additionally, 16 people (10.5%) experienced a terminal event without experiencing a non-terminal event. The average age of patients at the time of diagnosis was 55.6 (SD 13.1) years, with an age range of 21–84 years. The average age of diagnosis in patients with and without recurrence was 56.7 (13.4 SD) and 54.7 (12.8 SD) years, respectively.

The study found that the median survival of patients was 0.61 (95% confidence interval (CI): 42.2–79.8) months. Furthermore, the median survival for patients with recurrence was 47.0 (73 0.0–21.0) months. The median time from the non-terminal event to the terminal event was 10.0 (12.2 – 33.2) months, respectively. Table 1 shows the 1-, 3-, 5- and 10-year survival probabilities for the terminal event, the non-terminal event, and after disease recurrence.

**Table 1:** Survival probability for patients.

Measures	Recurrence outcome (%)	Death outcome (%)	Death after recurrence (%)
1-year	67.4	86.9	64.7
3-year	51.9	62.1	51.9
5-year	45.3	50.4	45.3
10-year	40.3	42.3	40.3

Furthermore, it is noteworthy that among patients with recurrence, 110 (84%) had developed metastases to other sites, highlighting the aggressive nature of the disease. Of those patients, 76 (58%) received more than six rounds of chemotherapy, indicating a possible correlation between the intensity of treatment and disease progression. It is also interesting to note that among patients who died during the study period, 94 (77.7%) had developed metastases to other sites, and 61 (50.4%) had received more than six rounds of chemotherapy. Additionally, 23 (19.0%) of those patients were in stage B, and 57 (47.1%) were in stage D, underscoring the importance of early detection and intervention in improving patient outcomes.

### PH assumption for Cox regression

The results of the log-rank test indicated that age at diagnosis was a significant predictor of survival (*P*-value < 0.05), with lower survival rates observed in patients over 70 years of age. Metastasis to other sites was also found to be a significant predictor of both terminal and non-terminal events (*P*-value < 0.05). Patients who received less than six chemotherapy sessions had a higher rate of events compared to those who received any chemotherapy. However, when the number of chemotherapy sessions was more than six, the outcome rate was decreased (*P* value = 0.057). The disease stage, PT stage, and PN stage were significant predictors of both terminal and non-terminal events (*P*-value < 0.05), with more advanced stages associated with a significant increase in the rate of both terminal and non-terminal events. Patients with advanced disease stages had lower survival rates for all three events.

### Results of multivariate Cox regression models for the non-terminal event (recurrence)

The study found that patients over the age of 70 had a higher risk of recurrence with a hazard ratio of 1.970 (95% CI = 3.204–1.212) compared to those below 50 years old. Metastasis to other sites was also significantly associated with an increased risk of recurrence (HR = 16.860, 95% CI = 27.756–10.241). Patients with disease stage D had a higher risk of recurrence compared to those at stage B (HR = 2.994, 95% CI = 1.757–5.101), as well as patients with PN stage at N2 compared to N0 (HR = 2.133, 95% CI = 1.233–3.690). The results of the Cox model for the non-terminal event were presented in Table 2, and the likelihood ratio confirmed the relationship between the set of variables (log likelihood = -536.485, LR chi2 (12) = 267.44, *p* > 0.000). According to Harrell's C index (= 0.86), there was a good match between the observed values and the predicted values.

**Table 2:** Multivariate Cox regression results for non-terminal event of recurrence.

Variables		HR	95% CI	*P*-value
Age at Diagnosis (years)	< 50	Referent	–	
	51–70	0.817	0.566–1.178	0.364
	> 70	1.970	1.211–3.205	0.022*
Metastasis to other sites	No	Referent	–	
	Yes	16.860	10.242–27.756	< 0.001*
Number of chemotherapies	No	Referent	–	
	< 6	1.133	0.631–2.035	0.725
	6 +	0.758	0.435–1.321	0.412
Grade differentiation level	Well	Referent	–	
	Moderate	1.006	0.707–1.431	0.978
	Poor	1.650	0.894–3.046	0.179
Disease stage	B	Referent	–	
	C	1.292	0.691–2.418	0.501
	D	2.993	1.757–5.101	0.001*
PN-Stage	N0	Referent	–	
	N1	1.110	0.681–1.809	0.726
	N2	2.133	1.233–3.690	0.023*
	NX	0.968	0.519–1.803	0.931

*HR*: Hazard ratio, *CI*: confidence interval.

*: *P*-value < 0.05.

### Results of multivariate Cox regression models for the terminal event (death)

The analysis revealed that metastasis to other organs was associated with an increased risk of death (HR = 5.628, 95% CI 3.533–8.965). However, the number of chemotherapy sessions greater than six was associated with a decrease in the risk of death (HR = 0.442, 95% CI: 0.258–0.756). Additionally, the disease stage at level D compared to level B was associated with an increased risk of death (HR = 2.748, 95% CI: 1.524–4.954), and the PN Stage at the N2 level compared to the N0 level was associated with an increased risk of death (HR = 2.303, 95% CI: 1.330–3.989).

Furthermore, the Cox model results presented for the final event 2 showed a good match between the observed values and predicted values, with a log likelihood of − 390.198 and LR chi2^7^ of 132.65 (*p* > 0.000) and Harrell's C index of 0.82 (Table 3).

**Table 3:** Multivariate Cox regression results for terminal event of death.

Variables		HR	95% CI	*P*–value
Metastasis to other sites	No	–	–	
	Yes	5.628	3.533–8.966	< 0.001*
Number of chemotherapies	No	–	–	
	< 6	1.048	0.602–1.826	0.888
	6 +	0.442	0.258–0.756	0.012
Disease stage	B	–	–	–
	C	1.826	0.955–3.489	0.126
	D	2.748	1.524–4.954	0.005*
PN-Stage	N0	–	–	–
	N1	1.306	0.801–2.128	0.369
	N2	2.303	1.330–3.989	0.012*
	NX	1.069	0.557–2.052	0.866

**HR: Hazard ratio.**

**CI: confidence interval.**

*: P–value < 0.05.

### Results of neural networks

In this study, we employed a specific neural network model known as the Multi-Layer Perceptron (MLP) for the purpose of data analysis. The MLP model underwent training with a varying range of hidden units, spanning from 5 to 7. During the training phase, a total of 20 networks were utilized, and subsequently, an additional 5 networks were employed for the retraining process.

**Figure 1:**
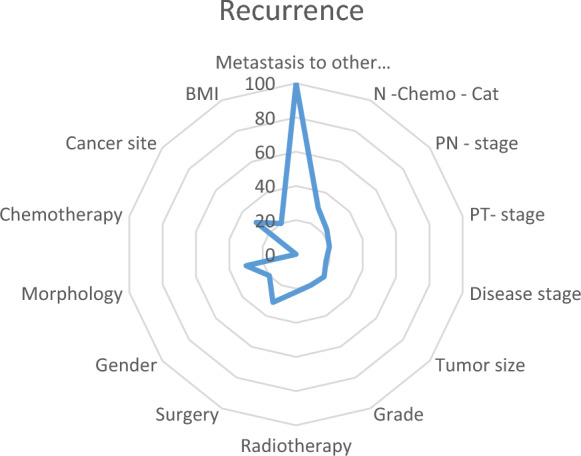
Radar plot for the best model selection for the recurrence outcome using the neural network model. *N—chemo-cat: Number of chemotherapies; Grade: Grade differentiation level.*

Upon evaluating the outcomes of the MLP model, we observed noteworthy findings regarding the factors influencing recurrence prediction. Notably, the presence of metastases to other anatomical sites emerged as the most significant predictor, with a substantial weight of 100%. This emphasizes the pronounced impact of metastatic occurrences on the likelihood of recurrence. Furthermore, the effect of surgical intervention was found to account for 31.177% of the predictive capacity, indicating its considerable contribution to recurrence prognosis. Likewise, the specific site of cancer manifestation demonstrated a discernible effect, contributing to 29.861% of the predictive power. These insights are visually represented in Fig. 1, which provides a comprehensive visualization of the relative weights associated with each predictor.

**Figure 2:**
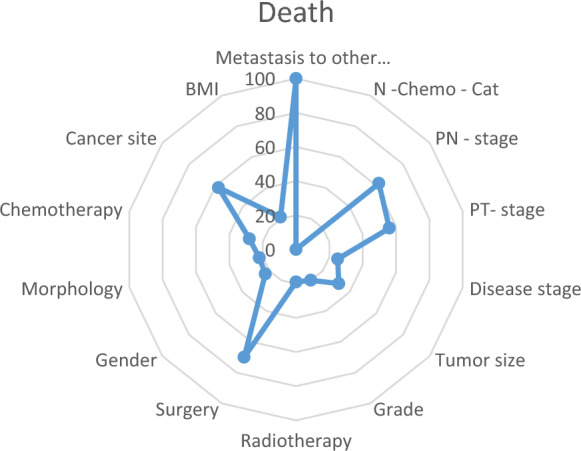
Radar plot for the best selected model for the death outcome using the neural network model. *N—chemo –cat: Number of chemotherapies; Grade: Grade differentiation level.*

Based on the results, it seems that metastasis to other sites was the most significant predictor of both recurrence and death. Surgery was also an important predictor of death, and PN-stage was a significant predictor of death (Fig. 2).

The sensitivity and specificity of the model for predicting recurrence were relatively high, with a sensitivity of 88.1% and a specificity of 83.7%. The positive predictive value (PPV) and negative predictive value (NPV) were also relatively high, with PPV at 84.1% and NPV at 87.8%. The area under the ROC curve was 0.86, which indicates good discrimination between the two classes. The accuracy was also high at 0.859.

For predicting death, the sensitivity and specificity were slightly lower than for predicting recurrence, with a sensitivity of 74.5% and a specificity of 83.3%. However, the PPV was high at 89.1%, while the NPV was lower at 64.1%. The area under the ROC curve was 0.79, indicating good discrimination between the two classes, and the accuracy was 0.776.

### Comparison of the Cox model and neural network model

The results based on the diagnostic indices, sensitivity, specificity, positive predictive value, negative predictive value, ROC curve, and accuracy for the Cox model, and the neural network model for the two variables of recurrence and death had shown in Tables 3 and 4. According to the results, the Cox model provides better results than the neural network model.

**Table 4:** Comparison of the Cox model and neural network model.

Outcome	Model	SE (95% CI)	SP (95% CI)	PPV (95% CI)	NPV (95% CI)	ROC area (95% CI)	Accuracy (%)
Death	Best of ANN	74.5 (61.0–85.0)	83.3 (65.3–94.4)	89.1 (76.4–96.4)	64.1 (47.2–78.8)	0.79 (0.70–0.88)	77
	Cox	73.6 (64.8–81.2)	89.6 (83.8–93.8)	84.0 (75.6–90.4)	82.0 (75.6–90.4)	0.82 (0.77–0.86)	88.6
Recurrence	Best of ANN	88.1 (74.4–96.0)	83.7 (69.3–93.4)	84.1 (69.9–93.4)	87.8 (73.8–95.9)	0.86 (0.78–0.93)	86
	Cox	85.5 (78.3–91.0)	98.0 (94.4–99.6)	97.4 (92.6–99.5)	88.8 (0.83–93.1)	0.92 (0.89–0.95)	91.9

*SE *  Sensitivity; *SP* Specificity; *PPV* Positive predictive value; *NPV* Negative predictive value; *ROC* Receiver operating characteristics; *CI* Confidence interval.

## Discussion

Our remarkable investigation assessed the efficacy of a neural network model and Cox regression in prognosticating early recurrence and death after curative surgery in 284 patients with resected colorectal cancer. Impressively, both models displayed impressive performance in predicting the aforementioned events, with significant predictors being metastasis to other sites, disease stage, and PN stage. Notably, patients aged above 70 were at a higher risk of recurrence compared to those below 50 years. Nonetheless, the Cox regression model outperformed the neural network model in terms of matching observed and predicted values.

These findings are consistent with previous studies that have been done in the field of predicting the survival of patients after surgery in colon cancer^22–24^. For instance, Wang et al. (2021) developed a nomogram to predict the overall survival of patients after curative surgery for colon cancer, which showed that age, tumor size, differentiation, TNM stage, and chemotherapy were independent predictors of overall survival^24^.

In a study conducted in 2022 to compare the accuracy of different machine learning models and Cox proportional hazards regression models for predicting the risk of dementia in people with mild cognitive impairment, the results showed that Cox regression models have a better predictive performance than more complex machine learning models^25^. Similarly, a 2021 study found that neural networks for predicting survival using clinical data may perform similarly to statistical methods such as the Cox model, but are often less adjusted^26^. In simpler cases, it may be better to use traditional statistical methods instead of neural networks^26^. Another study conducted in the field of urology to check the accuracy of predicting censored data using machine learning models and comparing it with the Cox regression model found that in all three datasets, the Cox regression model had acceptable predictions compared to machine learning models, including neural networks^27^.

A study conducted in 2023 developed a risk prediction model for chronic kidney disease (CKD) and its progression in patients with type 2 diabetes (T2D) using machine models and CoxPH model. The study concluded that the CoxPH model is the best method for predicting the incidence of CKD in T2D patients^11^. Also, In a study by Christodoulou et al. (2019)^28^, using a systematic approach, out of 71 studies that used logistic regression and machine learning models to predict binary outcomes, it was reported that there was a significant difference in the discriminative performance between the two methods The mentioned does not exist.

## Strengths and limitations

The study evaluating the efficacy of two different models for predicting early recurrence and death after curative surgery in patients with colorectal cancer has several strengths. Firstly, the study provides a comparison of two different models, a neural network model and Cox regression, for predicting these events, which can be useful for researchers and clinicians in deciding which model to use. Secondly, the study found significant predictors for both events, including metastasis to other sites, disease stage, and PN stage, which can be useful for future research and clinical practice. Lastly, the study's findings were consistent with previous research in the field, which increases their validity and reliability.

However, there are also several limitations to the study. Firstly, the study was limited to patients with resected colorectal cancer and may not be generalizable to other types of cancer or patients who did not have surgery. Future directions can be recommended to plan prospective studies in other cancer types and among all patients not only those had surgery. Secondly, the study did not include other potential predictors of early recurrence and death, such as comorbidities or lifestyle factors, which were not available in our database. Comprehensive studies with a complete dataset are strongly recommended to consider the effect of those variables in the models. These may affected the accuracy of the models.

## Conclusion

In conclusion, our study assessed the efficacy of two models, neural network and Cox regression, in prognosticating early recurrence and death after curative surgery in patients with resected colorectal cancer. Both models displayed impressive performance, with significant predictors including metastasis to other sites, disease stage, and PN stage. The study's findings were consistent with previous research in the field and highlight the importance of considering age as a potential predictor of recurrence risk. While the Cox regression model outperformed the neural network model in terms of matching observed and predicted values, other studies have found that neural networks may perform similarly to statistical methods such as the Cox model in certain cases. It is important for future research to consider potential interactions between predictors and assess the cost-effectiveness of using different models in clinical practice. Overall, our study provides valuable insights into the use of predictive models in oncology and underscores the need for further research in this area.

## Data Availability

Access to the data and materials that underlie the results of this study can be provided by MAJ, although certain limitations apply to their availability due to the licensing agreement for this study. As a result, the data are not publicly accessible. Nonetheless, interested parties may contact the authors for access to the data upon making a reasonable request to MAJ.
